# Antibiotic prescribing in Danish general practice in the elderly population from 2010 to 2017

**DOI:** 10.1080/02813432.2021.2004754

**Published:** 2021-11-24

**Authors:** Maria Louise Veimer Jensen, Rune Munck Aabenhus, Barbara Juliane Holzknecht, Lars Bjerrum, Jette Nygaard Jensen, Volkert Siersma, Gloria Córdoba

**Affiliations:** a Department of Clinical Microbiology, Copenhagen University Hospital - Herlev and Gentofte, Copenhagen, Denmark; b The Research Unit for General Practice and Section of General Practice, Department of Public Health, University of Copenhagen, Copenhagen, Denmark; c Department of Clinical Medicine, University of Copenhagen, Copenhagen, Denmark

**Keywords:** General practice, antibiotic prescriptions, prescriptions patterns, elderly population, aged

## Abstract

**Objective:**

This study aimed to describe prescription of antibiotics to the elderly population in general practice in Denmark from 2010–2017.

**Design:**

This is a national register-based observational study.

**Setting:**

General practice, Denmark

**Main outcome measure:**

The main outcome measure was prescriptions/1,000 inhabitants/day (PrID) in relation to year, age and sex, indication, and antibiotic agent.

**Subjects:**

In this study, we included inhabitants of Denmark, ≥65 years of age between 01st July 2010–30th June 2017.

**Results:**

A total of 5,168,878 prescriptions were included in the study. Antibiotic prescriptions decreased from 2.2 PrID to 1.7 (-26.9%, CI95% [-31.1;-22.4]) PrID during the study. The decrease in PrID was most noticeable among 65–74-year-olds (-25%). The ≥85-year-olds were exposed to twice as many PrID than the 65–74-year-olds, but only accounted for 20% of the total use. Urinary tract infection (UTI) was the most common indication for antibiotic prescription and increased with advancing age. The most commonly prescribed antibiotics were pivmecillinam and phenoxymethylpenicillin. Prescribing with no informative indication was present in one third of all cases.

**Conclusion:**

The prescription of antibiotics in the elderly population in general practice decreased from 2010 to 2017. The oldest age group was exposed twice as frequently to antibiotic prescriptions as the 65–74-year-olds. The smallest reduction was observed for the ≥85-year-olds, suggesting targeting interventions at this group.

**Key Points**
- High antibiotic use among elderly is well known and studies indicate mis- and overuse within this population. Our study shows.
- The prescription rate is decreasing within all age groups of the elderly population.
- The ≥85-year-olds receive twice as many prescriptions/1000/day as the 65–74-years-olds.

## Introduction

The unprecedented increase in antibacterial resistance rates is a serious threat to public health [1]. Antibiotics are among the most commonly prescribed drugs in general practice and as antibiotic use itself is the main driver of the rising antibacterial resistance [2,3], we need to promote a more judicious and rational prescribing approach to curb inappropriate antibiotic use. Such antimicrobial stewardship programs have gained increasing focus over the last decades [4]. Surveillance and monitoring of antibiotic use play a central role in stewardship programs as they enable the identification of nonrational use, intervention targets and facilitate the evaluation of interventions [5,6].

During the last decade, Denmark has seen a decrease in the use of antibiotics for all age groups, except for the elderly ≥80 years [7]. Current demographic projections in Europe show a trend towards the elderly, people ≥65 years of age, making up more of the population. By the year 2100, a projected 31% of the population will be ≥65 years of age, while those ≥80 years old will constitute almost 15% of the total European population [8].

The elderly population is at increased risk of many infectious diseases, including urinary tract infections (UTI) and respiratory tract infections (RTI), in turn exposing them to the consequences of antibacterial resistance, such as increased mortality and morbidity [9,10]. Furthermore, the elderly population is more susceptible to potential side effects of antibiotic treatments and these may occur more frequently in frail elderly with co-morbidities and exposure to polypharmacy [11–13].

The projected rise in the number of elderly in combination with the increase in infectious diseases requiring antibiotic treatment among this group, implies that it is important to understand current patterns and trends in prescriptions of antibiotics within this group. Unfortunately, detailed information on antibiotic use in the elderly population is still scarce. An international study from 2017 found that on a given day 10.5% of long-term health care facility residents in Denmark received antibiotic treatment [14]. This places Denmark, on par with Spain, as the highest antibiotic users in Europe in this population and far above average (4.9%). Aabenhus et al. [15] demonstrated that the use of antibiotics in general practice increases with the patients’ age, and that the elderly population is more often prescribed second line antibiotics. More detailed knowledge about consumption patterns and temporal trends are essential to identify areas where antibiotic use can be reduced or rationalized.

To this end, we conducted the present study to describe trends of antibiotic use by year, age group, clinical indication and antibiotic type among the elderly population in Denmark from 2010–2017.

## Material and methods

### Study design, population and data collection

This is a register-based observational study, describing antibiotic prescriptions in general practice among the elderly population in Denmark from 2010–2017. The study population was all Danish elderly aged ≥65 years or more. Data on antibiotic prescriptions were obtained from the Danish National Prescription Registry. This registry contains comprehensive and complete information on all reimbursed prescriptions redeemed by Danish inhabitants at outpatient pharmacies since 1995 [16]. The following inclusion criteria for prescriptions were used: redeemed by a patient ≥65 years of age; prescription of an antibacterial agent (Anatomical Therapeutic Chemical (ATC) classification system J01: antibacterial agent for systemic use and ATC P01AB01: metronidazole); date of reimbursement between 01st July 2010–30th June 2017. For each antibiotic prescription, the following variables were obtained: (a) clinical indication, (b) type of antibiotic, (c) dose, (d) date of purchase, (e) patient sex and age and (f) practice identification number.

### Data analysis

A dataset consisting of antibiotic prescriptions from general practice was constructed using the unique practice identification number.

### Outcome measure

The primary outcome measure for antibiotic prescriptions was calculated as the number of prescriptions/1,000 inhabitants/day (PrID). Results in Defined Daily Dose (DDD)/1,000 inhabitants/day (DID) are provided in supplementary materials. DDD is the average maintenance dose per day for a drug used for its main indication, as defined by the World Health Organization [17]. All results are available in Supplementary Table S1 + S2.

### Indications

In Denmark, the general practitioner (GP) must select a suitable clinical indication from a pre-specified drop-down list when prescribing an antibiotic treatment. If no suitable indication is available, the GP can enter a free text as indication, but this information is not available for data extraction. Until 2014 the GP could choose the indication ‘unspecified infection’ [18]. Based on these clinical indications, prescriptions were grouped according to anatomical site, namely respiratory tract infections including ear infections (RTI), urinary tract infections (UTI) and skin and soft tissue infections (SSTI). Indications not belonging to an anatomical site were grouped as ‘others’ together with central nervous system infections, genital infections, gastrointestinal infections and bone and joint infections (collectively constituting less than 5% of all prescriptions). Prescriptions labelled ‘unspecified infections’, missing indications or free text indications were grouped together as missing indication. A complete list of indications included in each group is available in Table S3. Prescriptions with indications obviously not associated with justifiable antibiotic prescribing such as ‘high blood pressure’ were excluded as these were regarded as a mistaken entry by the GP or system errors at pharmacy level. DDD values less than one per package were excluded. Observations with more than 10 redeemed packages at once were likewise excluded.

### Yearly measures

To account for varying seasonal changes in specific infectious diseases, a yearly measure was constructed from 01st July 2010–30th June 2011 and so forth, to ensure a complete winter season within each year. For these yearly measures, we used the specific population of elderly ≥65 years of Denmark on January 1st for each study year (e.g. January 1st, 2011 for the period 1st July 2010–30th June 2011) in the analyses.

### Age groups

The study population was divided into the three age groups; 65–74 years, 75–84 years and ≥85 years. When dividing the population according to age groups, the population size for the specific age group was used to calculate PrID and DID to enable inter-group comparisons. Data regarding population statistics was collected from www.statistikbanken.dk.

### Statistical analysis

Change over the study period in percentages was assessed in a linear regression of logarithm transformed PrID and DID on time. All data work was performed in SAS Software version 9.4 (SAS Institute Inc., Cary, NC, USA).

## Results

### General findings

From 2010 to 2017, the Danish elderly population increased by 17.3% from 933,781 to 1,095,172 individuals. The general population characteristics are shown in Table 1. The distribution of age groups and sex ratio remained stable during the study period.

**Table 1.** populations characteristics between 2010–2017.

Year	2010–2011	2011–2012	2012–2013	2013–2014	2014–2015	2015–2016	2016–2017
Population (No)	933,781	968,084	999,801	1,026,734	1,051,129	1,074,422	1,095,172
Age 65–74 years (%)	58	59	59	60	59	59	59
Age 75–84 years (%)	30	29	29	29	29	30	30
Ag*e* ≥ 85 years (%)	12	12	12	11	12	11	11
Female (%)	55	55	55	55	54	54	54

From 1st July 2010–30th June 2017, a total of 5,177,237 prescriptions were identified. After exclusion of prescriptions with indications not associated with antibiotic prescribing (*n* = 7,703), with DDD values < one per package (*n* = 384) and with > 10 redeemed packages (*n* = 272), 5,168,878 prescriptions were included in the study.

**Figure 1.**
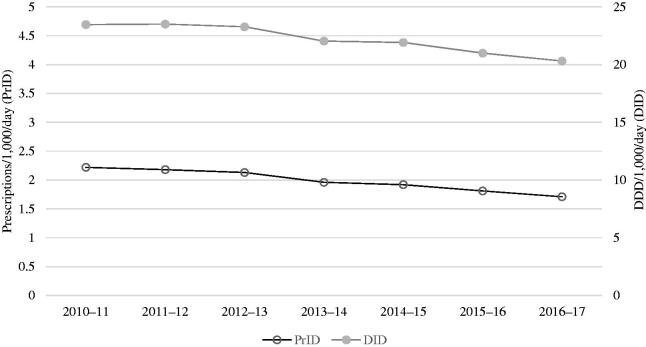
prescriptions/1,000/day (PrID) and DDD/1,000/day (DID) by year.

**Figure 2.**
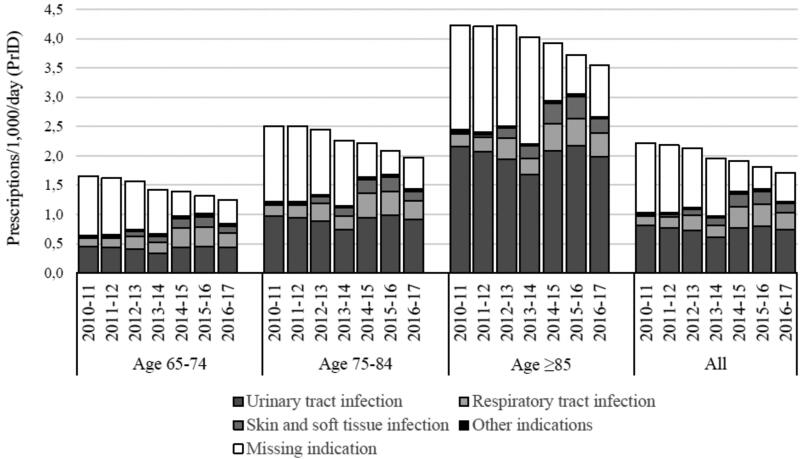
prescriptions/1,000/day by year, age group and indication.

PrID decreased from 2.2 to 1.7 (−26.9%, CI95% [−31.1; −22.4]), while DID decreased from 23.5 to 20.3 (−16%, CI95% [−21.0; −11.6]) during the study period (Figure 1). PrID decreased for all age groups but most pronounced in age group 65–74 (−25.0%) compared to −16.2% in age group ≥85-year-olds. While the decrease in age group 65–74 was similar in women (−25.0%) and men (−23.7%), the decrease in the oldest age group was more pronounced in women (−19.2%) than in men (−7.5%). From 2010 to 2017, the ≥85-year-old in average constituted 11% of the general elderly population but accounted for 23% of the total of antibiotic prescriptions, whereas the 65–74-year-olds constituted 60% of the general population but accounted for 43%. Patients in age group ≥85-year-olds received more than twice as many PrID than the 65–74-year-olds (Figure 2).

### Antibiotic treatment by indication

Figure 2 illustrates the differences in indications for antibiotic prescriptions during the study period and between age groups.

An increase in all specific indications was observed as missing indications decreased to approximately 30% over the study period. UTI constituted the majority of the available indications for all years and age groups. However, an absolute decrease in PrID for UTI was observed during the study. RTI increased relatively by 50% and absolutely by seven percentage points. This pattern was present in all age groups. Similarly, SSTI increased by eight percentage points.

UTI was the most prevalent indication in age group ≥85-year-olds across time accounting for 77.3% of all available indications (excluding ‘missing indication’), compared to 53.0% in the age group 65–74 years and 65.8% in 75–84 years. RTI and SSTI accounted for 29.3 and 13.1% of the available indications in the youngest age group, 21.8 and 10.0% in the 75–84-year-olds and 13.2 and 8.1% in the oldest age group.

### Antibiotic treatment by antibiotic type

**Figure 3.**
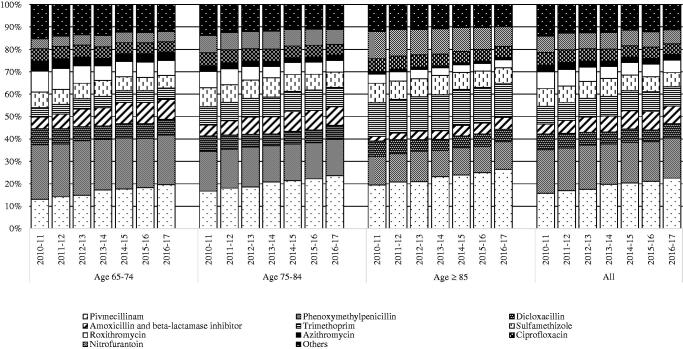
cumulative percentages of prescriptions/1,000/day by year, age group and antibiotic agent.

Figure 3 illustrates the cumulative percentual distribution of antibiotic agents prescribed to the Danish elderly population from 2010 to 2017.

In all, penicillin (J01C) constituted approximately 50% of the antibiotic consumption in the elderly population (pivmecillinam 19.0%, phenoxymethylpenicillin 18.5%, amoxicillin and beta-lactamase inhibitor 6.8% and dicloxacillin 6.1%). The relative consumption of this group of antibiotics increased for all age groups during the study period. This relative increase was due to an absolute increase in PrID of pivmecillinam and amoxicillin and beta-lactamase inhibitor and the overall absolute decrease in total PrID. Prescriptions of quinolones remained stable during the study, constituting approximately 5% in 2016–2017, in all age groups which corresponds to an actual decrease in the total amount of PrID from 0.13 to 0.09.

For the entire study period, the top three agents used for UTI made up 73.7% of all prescriptions labelled UTI; pivmecillinam (39.6%), sulfamethizole (16.9%) and trimethoprim (17.2%). Likewise, prescriptions labelled RTI were dominated by phenoxymethylpenicillin (38.2%), amoxicillin and beta-lactamase inhibitor (27.6%) and roxithromycin (14.3%) accounting together for 80.1% of this indication. During the study period a trend in prescriptions labelled RTI was noted where the proportion of phenoxymethylpenicillin decreased from 41.1% to 36.6%. Conversely, amoxicillin and beta-lactamase inhibitor increased from constituting 18.2% to constituting 30.8% of all prescriptions labelled RTI. For prescriptions labelled SSTI, the most frequently prescribed agents were phenoxymethylpenicillin (38.1%), dicloxacillin (36.1%) and flucloxacillin (8.2%) which together made up 82.4%. Prescriptions labelled Others were dominated by ciprofloxacin (32.9%), phenoxymethylpenicillin (20.4%) and dicloxacillin (16.4%).

## Discussion

### Statement of principal findings

In this study, we have characterized the prescriptions of antibiotics in general practice among the elderly population in Denmark from 2010–2017 by year, age group, clinical indication and antibiotic type.

PrID decreased from 2.2 to 1.7 over the seven-year study period. The ≥85-year-olds were exposed twice as frequently to antibiotic prescriptions as the 65–74-year-olds. This difference was likely driven by a higher number of UTI treatments in the ≥85-year-olds. A lower decrease was observed among men (−7.5%) than women (−19.2%) in age group ≥85-year-olds.

Throughout the study period, pivmecillinam and phenoxymethylpenicillin were the most prescribed antibiotic agents. In Denmark, pivmecillinam is generally first choice treatment for UTIs and phenoxymethylpenicillin for most RTIs. As UTI and RTI was the most common indications, this indicate that the choice of antibiotic type is based on current guidelines. Furthermore, the top three antibiotic agents prescribed for UTI, RTI and SSTI accounted for 73.7%, 80.1% and 82.4% of the total amount of prescriptions respectively, indicating that GPs tend to prescribe the same type of antibiotics for the same indications. Fluoroquinolones are decreasingly used in Danish general practice and made up only approximately 5% of the total use. Worryingly, a slight increase in prescription of amoxicillin with beta-lactamase inhibitor was noted, as this is on the watch list in WHO AWaRe index.

### Strengths and weaknesses of the study

Certain strengths and limitations of the study need to be addressed. First, we chose to primarily report the results in PrID, as this unit is perceived more clinically understandable. Some clinicians may only have vague references for DDD or DID values, compared to PrID. Furthermore, when using prescriptions and PrID as measurements we circumvent the inconvenience of changing guidelines for antibiotic treatment, package sizes and strength of tablets, which may influence the value of DDD, and subsequently DID. On the other hand, when using PrID and DID one does not take treatment failure into account and may therefore not precisely reflect clinical treatment episodes. However, as we set out to examine prescription of antibiotics, we found that PrID was the most sensible measurement.

The nationwide scope of the project is a clear advantage as the Danish national registers provide an opportunity to examine almost the entire use of antibiotics in general practice. In Denmark, antibiotics for systemic use are only available by prescription and all redeemed prescriptions are registered in the prescription database, which makes the database complete. While the database is complete in this regard, one-third (2016–2017) of the prescriptions did not have an informative clinical indication. As noted by Aabenhus et al. [18], it is important that indications of this registry are validated before widespread use. Unfortunately, it is for now not possible to merge the GP’s different journal systems to the Danish prescription database, both by law and practicality. During our study period the amount of missing indications decreased from more than 50% to around 30%. A study from UK also showed a rate of 31% of non-informative/missing indications for antibiotic prescriptions [19]. Certain groups may be overrepresented in this group of treatments with the non-informative indication. Besides that, the decreasing percentage of missing indications and subsequent shift in indications for antibiotic prescriptions during the present study may not reflect a change in actual infectious diseases and/or antibiotic treatment, but a change in documentational habits of the GP. Furthermore, given that many episodes of asymptomatic bacteriuria (ASB) are managed as UTIs, the physician's selected indication does not necessarily reflect this actual condition.

### Findings in relation to other studies

In line with DANMAP [20], our study has reported a decrease in antibiotic prescription. However, the prescription rate among the elderly is still well above the general population average (20.3 vs. 13.4 DID) [21]. The drift in antibiotic indications toward UTI with increasing age is comparable to trends observed in Sweden [22]. A US study from 2018 by Kabbani et al. [23] describing outpatient antibiotic use among the elderly population from 2010–2014 found that quinolones constituted 24% of all prescribed antibiotic for the elderly compared to 5.5% in our study (2010–17). The difference may be due to very different health care systems and populations but more importantly, Danish guidelines specify only to use quinolones when microbiological susceptibility testing support this and no other options are available.

The present decrease in the antibiotic prescription rate is concurrent with a slight, but significant decrease in the resistance rates of E. coli from urine samples in primary health care. This could lead to less treatment failure and hence lower the antibiotic prescribing rate, but as the general resistance rate in Denmark is low [20], this may not be the main driver for the observed decrease. There have not been any major changes in treatment guidelines during the study period.

In the Danish National Action plan from 2017 [24], a national target was set at 350 prescriptions/1,000/year (≈0.96 PrID) by 2020. Although the total antibiotic use declined significantly during the study period it is evident that in the elderly population, we are still far from reaching this goal. In 2016–2017 the elderly population used approximately 619 treatments/1,000/year (≈1.7 PrID). Likewise, in Sweden, which is known for a prudent use of antibiotics, the elderly population has a substantial higher use than the national target. To curb this higher use in the elderly population several Danish initiatives have been launched, for example, specific “nursing home doctors”, focus on increasing knowledge of infections and antibiotic treatments among nursing home staff [25] and improving diagnostic tools for UTIs in nursing homes [26].

When comparing the rate of antibiotic prescriptions in the different age groups, it was evident that the ≥85-year-olds had a very high use of antibiotics. The reason for this is unknown but may lie in the considerably higher proportion of institutionalized individuals, for which a high use of antibiotics is well known. Within the group of institutionalized individuals, UTI is a central driver of the high prescription rate due to the following: high incidence of UTI, high prevalence of ASB [27], difficulties in the diagnostic process of UTI and potential atypical presentation. The difficulties with UTI diagnostics and ASB (and treatment of this) may be obvious targets for intervention as no methodological strong evidence illustrates an association between unspecific symptoms and UTI or the benefit of treatment of ASB [28–30].

Furthermore, infectious diseases in this group is more often accessed and communicated second hand through care takers, which can complicate the diagnostic process and hence treatment decision.

### Meaning of the study

Finally, this study enables the identification of patterns of antibiotic use within the elderly population and proposes potential interventions aiming to reduce and rationalize antibiotic use. This study also indicates that the interventions already implemented in Denmark (mainly national campaigns, focus on rational pharmacotherapy and the national council of antibiotic stewardship) has been somewhat successful. However, it is important to remember that antibiotic treatment is sometimes of great importance for especially frail elderly. Knowledge on how to reduce antibiotic use without compromising the well-being of these individuals is warranted.

In summary, the total antibiotic use in PrID declined from 2010–2017 in the elderly population in general practice, Denmark. This decrease was observed in all age groups, but not evenly distributed. The rate of antibiotic prescriptions increased with older age. This was especially the case for UTI treatments. The largest proportion of antibiotic prescriptions were prescribed to the 65–74-year-olds due to the larger population size of this group. Interventions to reduce unnecessary antibiotic use among the oldest and to reduce overprescribing for UTI are two possible relevant targets to effectively lower antibiotic use in the future.

## Supplementary Material

Supplemental Material
