# Supplementary material for: Antibiotic prescribing in Danish general practice in the elderly population from 2010 to 2017
Source: Scand J Prim Health Care. 2021 Nov 24;39(4):498–505. doi: 10.1080/02813432.2021.2004754 (PMC8725860; doi:10.1080/02813432.2021.2004754)
Supplement: Supplemental Material [file IPRI_A_2004754_SM2693.docx]

## Table S1: Antibiotic prescriptions expressed as prescriptions/1,000/day (PrID) and DDD/1,000/day (DID) from 2010–2017 by indication and age group

|  |  | Prescriptions/1,000/day (PrID) | | | | | | DDD/1,000/day (DID) | | | | | | |
| --- | --- | --- | --- | --- | --- | --- | --- | --- | --- | --- | --- | --- | --- | --- |
| Year | Indication | All | Age 65–74 | | Age 75–84 | | Age ≥85 | All | | Age 65–74 | Age 75–84 | | | Age ≥85 |
| 2010–11 | UTI | 0.81 | | 0.45 | 0.97 | 2.16 | | 8.57 | 4.62 | | | 10.51 | 22.93 | |
|  | RTI | 0.17 | 0.15 | | 0.19 | | 0.21 | 1.88 | | 1.67 | 2.13 | | | 2.27 |
|  | SSTI | 0.02 | 0.02 | | 0.03 | | 0.04 | 0.30 | | 0.28 | 0.31 | | | 0.43 |
|  | Others | 0.03 | 0.03 | | 0.03 | | 0.03 | 0.29 | | 0.27 | 0.28 | | | 0.38 |
|  | Missing | 1.19 | 1.01 | | 1.29 | | 1.78 | 12.43 | | 10.67 | 13.71 | | | 17.77 |
|  | Total | 2.22 | 1.66 | | 2.51 | | 4.22 | 23.46 | | 17.51 | 26.94 | | | 43.78 |
| 2011–12 | UTI | 0.77 | 0.43 | | 0.95 | | 2.07 | 8.32 | | 4.56 | 10.37 | | | 22.16 |
|  | RTI | 0.19 | 0.16 | | 0.21 | | 0.24 | 2.07 | | 1.82 | 2.38 | | | 2.59 |
|  | SSTI | 0.04 | 0.03 | | 0.04 | | 0.06 | 0.41 | | 0.35 | 0.45 | | | 0.62 |
|  | OTHERS | 0.03 | 0.03 | | 0.03 | | 0.03 | 0.28 | | 0.27 | 0.27 | | | 0.33 |
|  | MISSING | 1.16 | 0.97 | | 1.28 | | 1.81 | 12.41 | | 10.47 | 13.90 | | | 18.46 |
|  | Total | 2.18 | 1.62 | | 2.50 | | 4.21 | 23.50 | | 17.47 | 27.37 | | | 44.16 |
| 2012–13 | UTI | 0.72 | 0.41 | | 0.89 | | 1.94 | 7.73 | | 4.29 | 9.66 | | | 20.63 |
|  | RTI | 0.26 | 0.22 | | 0.30 | | 0.35 | 2.92 | | 2.50 | 3.4 | | | 3.92 |
|  | SSTI | 0.10 | 0.08 | | 0.12 | | 0.18 | 1.12 | | 0.94 | 1.23 | | | 1.77 |
|  | OTHERS | 0.03 | 0.03 | | 0.03 | | 0.03 | 0.35 | | 0.35 | 0.36 | | | 0.36 |
|  | MISSING | 1.01 | 0.82 | | 1.12 | | 1.71 | 11.15 | | 9.15 | 12.50 | | | 18.11 |
|  | Total | 2.13 | 1.57 | | 2.45 | | 4.22 | 23.27 | | 17.23 | 27.15 | | | 44.79 |
| 2013–14 | UTI | 0.61 | 0.34 | | 0.74 | | 1.69 | 6.46 | | 3.64 | 7.98 | | | 17.41 |
|  | RTI | 0.21 | 0.18 | | 0.24 | | 0.27 | 2.43 | | 2.12 | 2.82 | | | 3.03 |
|  | SSTI | 0.13 | 0.10 | | 0.14 | | 0.21 | 1.39 | | 1.17 | 1.52 | | | 2.26 |
|  | OTHERS | 0.03 | 0.03 | | 0.03 | | 0.03 | 0.40 | | 0.41 | 0.39 | | | 0.39 |
|  | MISSING | 0.98 | 0.76 | | 1.11 | | 1.83 | 11.35 | | 8.79 | 12.97 | | | 20.7 |
|  | Total | 1.96 | 1.42 | | 2.27 | | 4.03 | 22.03 | | 16.13 | 25.68 | | | 43.79 |
| 2014–15 | UTI | 0.77 | 0.44 | | 0.95 | | 2.08 | 8.47 | | 4.79 | 10.51 | | | 22.71 |
|  | RTI | 0.37 | 0.32 | | 0.41 | | 0.46 | 4.28 | | 3.75 | 4.93 | | | 5.39 |
|  | SSTI | 0.21 | 0.17 | | 0.23 | | 0.35 | 2.32 | | 1.92 | 2.55 | | | 3.81 |
|  | OTHERS | 0.05 | 0.05 | | 0.04 | | 0.05 | 0.54 | | 0.54 | 0.52 | | | 0.56 |
|  | MISSING | 0.53 | 0.41 | | 0.58 | | 0.98 | 6.31 | | 5.00 | 7.04 | | | 11.31 |
|  | Total | 1.92 | 1.39 | | 2.22 | | 3.92 | 21.91 | | 16.00 | 25.55 | | | 43.78 |
| 2015–16 | UTI | 0.81 | 0.46 | | 0.98 | | 2.17 | 9.17 | | 5.13 | 11.37 | | | 24.9 |
|  | RTI | 0.37 | 0.32 | | 0.41 | | 0.46 | 4.31 | | 3.79 | 4.93 | | | 5.42 |
|  | SSTI | 0.22 | 0.18 | | 0.24 | | 0.37 | 2.51 | | 2.09 | 2.72 | | | 4.18 |
|  | OTHERS | 0.05 | 0.05 | | 0.04 | | 0.04 | 0.59 | | 0.61 | 0.56 | | | 0.53 |
|  | MISSING | 0.37 | 0.30 | | 0.40 | | 0.67 | 4.42 | | 3.63 | 4.87 | | | 7.41 |
|  | Total | 1.81 | 1.31 | | 2.09 | | 3.72 | 20.99 | | 15.25 | 24.45 | | | 42.44 |
| 2016–17 | UTI | 0.75 | 0.43 | | 0.91 | | 1.98 | 8.73 | | 4.97 | 10.77 | | | 23.37 |
|  | RTI | 0.29 | 0.25 | | 0.33 | | 0.40 | 3.50 | | 3.00 | 4.01 | | | 4.8 |
|  | SSTI | 0.15 | 0.12 | | 0.16 | | 0.26 | 1.85 | | 1.56 | 1.98 | | | 3.01 |
|  | OTHERS | 0.04 | 0.04 | | 0.03 | | 0.03 | 0.47 | | 0.49 | 0.44 | | | 0.43 |
|  | MISSING | 0.49 | 0.40 | | 0.54 | | 0.87 | 5.76 | | 4.71 | 6.40 | | | 9.64 |
|  | Total | 1.71 | 1.24 | | 1.97 | | 3.54 | 20.30 | | 14.73 | 23.60 | | | 41.25 |
|  |  |  |  | |  | |  |  | |  |  | | |  |

## Table S2: Type of antibiotic prescribed to the elderly population from 2010–17 expressed as prescriptions/1,000/day (PrID) and DDD/1,000/day (DID).

|  |  | Prescriptions/1,000/day (PrID) | | | | DDD/1,000/day | | | |
| --- | --- | --- | --- | --- | --- | --- | --- | --- | --- |
|  |  | All | Age 65–74 | Age 75–84 | Age ≥85 | All | Age 65–74 | Age 75–84 | Age ≥85 |
| Year | Antibiotic agent |  |  |  |  |  |  |  |  |
| 2010–11 | Phenoxymethylpenicillin | 0.43 | 0.40 | 0.44 | 0.53 | 4.35 | 4.09 | 4.46 | 5.31 |
|  | Pivmecillinam | 0.35 | 0.22 | 0.42 | 0.83 | 4.04 | 2.47 | 4.86 | 9.67 |
|  | Trimethoprim | 0.18 | 0.07 | 0.20 | 0.63 | 2.02 | 0.96 | 2.61 | 6.28 |
|  | Sulfamethizole | 0.18 | 0.12 | 0.21 | 0.37 | 0.54 | 0.35 | 0.65 | 1.13 |
|  | Nitrofurantoin | 0.16 | 0.07 | 0.19 | 0.51 | 1.89 | 0.97 | 2.38 | 5.15 |
|  | Roxithromycin | 0.16 | 0.15 | 0.18 | 0.17 | 1.58 | 1.48 | 1.73 | 1.67 |
|  | Dicloxacillin | 0.16 | 0.12 | 0.17 | 0.29 | 1.59 | 1.19 | 1.80 | 3.05 |
|  | Ciprofloxacin | 0.13 | 0.09 | 0.15 | 0.24 | 1.02 | 0.77 | 1.18 | 1.85 |
|  | Amoxicillin and beta–lactamase inhibitor | 0.10 | 0.09 | 0.12 | 0.09 | 1.51 | 1.33 | 1.92 | 1.35 |
|  | Azithromycin | 0.07 | 0.07 | 0.07 | 0.05 | 0.39 | 0.41 | 0.38 | 0.26 |
|  | Others | 0.31 | 0.25 | 0.35 | 0.51 | 4.53 | 3.51 | 4.96 | 8.05 |
|  | Total | 2.22 | 1.66 | 2.51 | 4.22 | 23.46 | 17.51 | 26.94 | 43.78 |
| 2011–12 | Phenoxymethylpenicillin | 0.41 | 0.38 | 0.43 | 0.53 | 4.20 | 3.89 | 4.36 | 5.33 |
|  | Pivmecillinam | 0.37 | 0.23 | 0.45 | 0.88 | 4.45 | 2.72 | 5.46 | 10.61 |
|  | Trimethoprim | 0.18 | 0.07 | 0.20 | 0.63 | 2.04 | 0.96 | 2.57 | 6.18 |
|  | Sulfamethizole | 0.16 | 0.11 | 0.20 | 0.34 | 0.50 | 0.33 | 0.61 | 1.06 |
|  | Nitrofurantoin | 0.16 | 0.08 | 0.19 | 0.50 | 1.88 | 0.99 | 2.40 | 5.08 |
|  | Roxithromycin | 0.16 | 0.15 | 0.18 | 0.17 | 1.54 | 1.43 | 1.69 | 1.69 |
|  | Dicloxacillin | 0.14 | 0.11 | 0.16 | 0.26 | 1.47 | 1.12 | 1.67 | 2.71 |
|  | Ciprofloxacin | 0.12 | 0.09 | 0.14 | 0.24 | 0.98 | 0.74 | 1.18 | 1.85 |
|  | Amoxicillin and beta–lactamase inhibitor | 0.13 | 0.11 | 0.16 | 0.13 | 1.95 | 1.68 | 2.50 | 1.95 |
|  | Azithromycin | 0.07 | 0.07 | 0.07 | 0.05 | 0.39 | 0.41 | 0.40 | 0.27 |
|  | Others | 0.28 | 0.23 | 0.31 | 0.47 | 4.10 | 3.20 | 4.52 | 7.43 |
|  | Total | 2.18 | 1.62 | 2.50 | 4.21 | 23.50 | 17.47 | 27.37 | 44.16 |
| 2012–13 | Phenoxymethylpenicillin | 0.42 | 0.38 | 0.44 | 0.57 | 4.27 | 3.91 | 4.45 | 5.70 |
|  | Pivmecillinam | 0.37 | 0.23 | 0.46 | 0.88 | 4.55 | 2.80 | 5.62 | 10.91 |
|  | Trimethoprim | 0.18 | 0.07 | 0.20 | 0.65 | 0.07 | 0.99 | 2.60 | 6.37 |
|  | Sulfamethizole | 0.16 | 0.11 | 0.20 | 0.34 | 0.49 | 0.32 | 0.61 | 1.05 |
|  | Nitrofurantoin | 0.15 | 0.07 | 0.19 | 0.47 | 1.76 | 0.94 | 2.27 | 4.72 |
|  | Roxithromycin | 0.14 | 0.12 | 0.15 | 0.17 | 1.31 | 1.18 | 1.43 | 1.65 |
|  | Dicloxacillin | 0.12 | 0.10 | 0.14 | 0.22 | 1.26 | 1.00 | 1.39 | 2.32 |
|  | Ciprofloxacin | 0.12 | 0.08 | 0.14 | 0.24 | 0.95 | 0.71 | 1.09 | 1.86 |
|  | Amoxicillin and beta–lactamase inhibitor | 0.15 | 0.12 | 0.19 | 0.16 | 2.31 | 1.95 | 2.96 | 2.52 |
|  | Azithromycin | 0.06 | 0.06 | 0.06 | 0.04 | 0.35 | 0.37 | 0.36 | 0.26 |
|  | Others | 0.27 | 0.21 | 0.30 | 0.47 | 5.95 | 3.07 | 4.37 | 7.44 |
|  | Total | 2.13 | 1.57 | 2.45 | 4.22 | 23.27 | 17.23 | 27.15 | 44.79 |
| 2013–14 | Phenoxymethylpenicillin | 0.35 | 0.32 | 0.37 | 0.47 | 3.66 | 3.37 | 3.78 | 4.85 |
|  | Pivmecillinam | 0.39 | 0.25 | 0.47 | 0.94 | 4.86 | 3.02 | 5.92 | 11.87 |
|  | Trimethoprim | 0.17 | 0.07 | 0.20 | 0.65 | 2.03 | 0.97 | 2.62 | 6.37 |
|  | Sulfamethizole | 0.16 | 0.10 | 0.20 | 0.35 | 0.42 | 0.31 | 0.61 | 1.11 |
|  | Nitrofurantoin | 0.14 | 0.07 | 0.18 | 0.45 | 1.73 | 0.92 | 2.21 | 4.70 |
|  | Roxithromycin | 0.10 | 0.09 | 0.12 | 0.13 | 1.01 | 0.91 | 1.12 | 1.31 |
|  | Dicloxacillin | 0.11 | 0.08 | 0.12 | 0.20 | 1.12 | 0.88 | 1.22 | 2.06 |
|  | Ciprofloxacin | 0.10 | 0.07 | 0.13 | 0.21 | 0.87 | 0.63 | 1.03 | 1.68 |
|  | Amoxicillin and beta–lactamase inhibitor | 0.14 | 0.12 | 0.18 | 0.16 | 2.14 | 1.81 | 2.71 | 2.39 |
|  | Azithromycin | 0.05 | 0.05 | 0.05 | 0.04 | 0.31 | 0.31 | 0.31 | 0.24 |
|  | Others | 0.25 | 0.20 | 0.27 | 0.44 | 3.88 | 2.99 | 4.13 | 7.21 |
|  | Total | 1.96 | 1.42 | 2.27 | 4.03 | 22.03 | 16.13 | 25.68 | 43.79 |
| 2014–15 | Phenoxymethylpenicillin | 0.35 | 0.32 | 0.36 | 0.48 | 3.67 | 3.35 | 3.79 | 5.02 |
|  | Pivmecillinam | 0.39 | 0.25 | 0.48 | 0.93 | 4.94 | 3.09 | 6.04 | 11.96 |
|  | Trimethoprim | 0.17 | 0.07 | 0.20 | 0.63 | 2.06 | 0.98 | 2.61 | 6.43 |
|  | Sulfamethizole | 0.13 | 0.09 | 0.17 | 0.30 | 0.37 | 0.27 | 0.52 | 0.94 |
|  | Nitrofurantoin | 0.13 | 0.06 | 0.16 | 0.41 | 1.57 | 0.84 | 1.98 | 4.37 |
|  | Roxithromycin | 0.11 | 0.09 | 0.12 | 0.13 | 1.03 | 0.92 | 1.14 | 1.31 |
|  | Dicloxacillin | 0.11 | 0.09 | 0.13 | 0.20 | 1.27 | 1.01 | 1.42 | 2.29 |
|  | Ciprofloxacin | 0.10 | 0.07 | 0.12 | 0.20 | 0.81 | 0.59 | 0.96 | 1.56 |
|  | Amoxicillin and beta–lactamase inhibitor | 0.16 | 0.13 | 0.20 | 0.19 | 2.41 | 2.03 | 3.02 | 2.85 |
|  | Azithromycin | 0.05 | 0.05 | 0.05 | 0.04 | 0.33 | 0.34 | 0.34 | 0.25 |
|  | Others | 0.22 | 0.17 | 0.25 | 0.40 | 3.45 | 2.59 | 3.75 | 6.80 |
|  | Total | 1.92 | 1.39 | 2.22 | 3.92 | 21.91 | 16.00 | 25.55 | 43.78 |
| 2015–16 | Phenoxymethylpenicillin | 0.32 | 0.29 | 0.33 | 0.43 | 3.40 | 3.11 | 3.55 | 4.54 |
|  | Pivmecillinam | 0.38 | 0.24 | 0.46 | 0.93 | 4.91 | 3.02 | 5.98 | 12.17 |
|  | Trimethoprim | 0.16 | 0.07 | 0.19 | 0.59 | 1.96 | 0.91 | 2.48 | 6.22 |
|  | Sulfamethizole | 0.12 | 0.08 | 0.15 | 0.27 | 0.33 | 0.23 | 0.46 | 0.85 |
|  | Nitrofurantoin | 0.12 | 0.06 | 0.15 | 0.36 | 1.42 | 0.77 | 1.79 | 3.85 |
|  | Roxithromycin | 0.10 | 0.09 | 0.11 | 0.13 | 0.99 | 0.88 | 1.09 | 1.25 |
|  | Dicloxacillin | 0.10 | 0.09 | 0.12 | 0.20 | 1.25 | 0.99 | 1.38 | 2.36 |
|  | Ciprofloxacin | 0.09 | 0.06 | 0.11 | 0.20 | 0.76 | 0.55 | 0.90 | 1.54 |
|  | Amoxicillin and beta–lactamase inhibitor | 0.15 | 0.12 | 0.18 | 0.19 | 2.30 | 1.93 | 2.86 | 2.84 |
|  | Azithromycin | 0.05 | 0.05 | 0.05 | 0.04 | 0.35 | 0.36 | 0.35 | 0.27 |
|  | Others | 0.22 | 0.16 | 0.23 | 0.38 | 3.32 | 2.51 | 3.61 | 6.54 |
|  | Total | 1.81 | 1.31 | 2.09 | 3.72 | 20.99 | 15.25 | 24.45 | 42.44 |
| 2016–17 | Phenoxymethylpenicillin | 0.31 | 0.27 | 0.32 | 0.44 | 3.23 | 2.92 | 3.35 | 4.53 |
|  | Pivmecillinam | 0.39 | 0.25 | 0.47 | 0.94 | 4.95 | 3.08 | 5.98 | 12.21 |
|  | Trimethoprim | 0.15 | 0.06 | 0.17 | 0.54 | 1.87 | 0.89 | 2.33 | 5.91 |
|  | Sulfamethizole | 0.11 | 0.07 | 0.13 | 0.24 | 0.33 | 0.21 | 0.42 | 0.76 |
|  | Nitrofurantoin | 0.11 | 0.06 | 0.13 | 0.31 | 1.24 | 0.70 | 1.55 | 3.29 |
|  | Roxithromycin | 0.09 | 0.08 | 0.10 | 0.13 | 0.94 | 0.83 | 1.04 | 1.26 |
|  | Dicloxacillin | 0.11 | 0.09 | 0.12 | 0.20 | 1.39 | 1.13 | 1.54 | 2.42 |
|  | Ciprofloxacin | 0.08 | 0.06 | 0.10 | 0.18 | 0.68 | 0.49 | 0.79 | 1.39 |
|  | Amoxicillin and beta–lactamase inhibitor | 0.13 | 0.11 | 0.16 | 0.19 | 2.20 | 1.79 | 2.69 | 3.02 |
|  | Azithromycin | 0.05 | 0.05 | 0.05 | 0.04 | 0.36 | 0.36 | 0.38 | 0.27 |
|  | Others | 0.19 | 0.15 | 0.22 | 0.35 | 3.11 | 2.34 | 3.53 | 6.20 |
|  | Total | 1.71 | 1.24 | 1.97 | 3.54 | 20.30 | 14.73 | 23.60 | 41.25 |

## Table S3: list of indications included in each indication group

| Indication group | Code | Description | Indication group | Code | Description |
| --- | --- | --- | --- | --- | --- |
| UTI | | | OTHERS | | |
|  | 0000103 | against cystitis |  | 0000479 | sepsis |
|  | 0000104 | against urinary tract infection |  | 0000486 | for prevention of serious infection |
|  | 0000105 | bladder pain |  | 0000125 | treatment and eradication of staphylococcus |
|  | 0000106 | pain with urinating |  | 0000131 | against tuberculosis |
|  | 0000126 | chronic urinary tract infection |  | 0000425 | against Lyme disease, stage II and III |
|  | 0000265 | for the prevention of urinary tract infection |  | 0000454 | against borrelia infection |
|  | 0000448 | against pelvic inflammation |  | 0000455 | prophylaxis after animal or human bites |
| RTI (incl. ear infections) | | |  | 0000794 | for prophylaxis before surgery |
|  | 0000122 | against sore throat |  | 0000484 | for prophylaxis before surgery |
|  | 0000123 | against pneumonia |  |  |  |
|  | 0000127 | +against bronchitis |  | 0000003 | against stomach ulcers |
|  | 0000197 | against sinusitis |  | 0000024 | against inflammation of the intestine |
|  | 0000325 | in lung disease |  | 0000026 | against diarrhea |
|  | 0000332 | against lung infection |  | 0000249 | against inflammation of the colon |
|  | 0000433 | lung infection in cystic fibrosis |  | 0000465 | against peptic ulcer (eradication of H. pylori) |
|  | 0000462 | against whooping cough |  | 0000717 | infection of the anal canal |
|  | 0000463 | against scarlet fever |  |  |  |
|  | 0000464 | Against the exacerbation of chronic obstructive pulmonary disease |  | 0000083 | vaginal infection |
|  | 0000481 | against epiglottitis |  | 0000085 | against yeast infection |
|  | 0000740 | acute exacerbation of chronic bronchitis |  | 0000290 | vaginal bacterial infection |
|  | 0000771 | acute exacerbation of chronic bronchitis |  | 0000450 | against inflammation of the vagina |
|  |  |  |  | 0000451 | against chlamydia/mycoplasma infection |
|  | 0000121 | against otitis media |  | 0000452 | against gonorrhea |
|  | 0000191 | eczema of the ear canal |  | 0000453 | against syphilis |
|  | 0000192 | against inflammation of the ear canal |  | 0000446 | against inflammation of the epididymis |
|  | 0000342 | against sputum from ear |  | 0000445 | against inflammation of the abdomen |
| SSTI | | |  | 0000447 | against inflammation of the urethra |
|  | 0000128 | against impure skin |  | 0000449 | against inflammation of the bladder neck gland |
|  | 0000224 | against infections of the skin |  |  |  |
|  | 0000225 | against genital warts |  | 0000132 | prevention of meningitis |
|  | 0000227 | against skin disorders |  | 0000296 | prevention of meningitis |
|  | 0000228 | against skin condition |  | 0000426 | against meningitis |
|  | 0000230 | against infected eczema |  | 0000482 | intracerebral abscess |
|  | 0000231 | against psoriasis with infection |  |  |  |
|  | 0000234 | against acne |  | 0000185 | iridocyclitis |
|  | 0000235 | severe acne |  | 0000186 | against eye disease |
|  | 0000236 | severe acne |  | 0000187 | chronic eye infection |
|  | 0000246 | infected psoriasis |  | 0000298 | against eye inflammation |
|  | 0000360 | against inflammation of the skin |  | 0000308 | against vernal eye infection |
|  | 0000409 | skin and soft tissue infections by gram positive bacteria |  | 0000470 | uveitis |
|  | 0000417 | against skin and soft tissue infection |  | 0000647 | infection of the cornea |
|  | 0000457 | against childhood ulcers |  | 0000651 | post-operative eye infection |
|  | 0000458 | against wound infection |  |  |  |
|  | 0000459 | against erysipelas |  | 000427 | against bacterial infection in bones and joints |
|  | 0000444 | against inflammation of the abdominal cavity |  |  |  |
|  | 0000456 | against mastitis | Missing/non informative indication | | |
|  | 0000460 | against heart valve inflammation |  | 0000084 | Against infection |
|  | 0000461 | for prevention of heart valve inflammation |  | 0000000 | Free text |
|  | 0000480 | abdominal cavity infection |  | Missing |  |
|  | 0000483 | against intra-abdominal infection |  | 0000312 | Against inflammation |
|  |  |  |  | 0000124 | Severe infection |
